# Validity of the Movement Behavior Questionnaire-Child (MBQ-C) Physical Activity Domain among Toddlers

**DOI:** 10.21203/rs.3.rs-8139126/v1

**Published:** 2025-12-22

**Authors:** Soyang Kwon, Nidhi S Gopagani, Isabella R Zylka, Sarah B Welch

**Affiliations:** Northwestern University; Northwestern University; Northwestern University; Northwestern University

**Keywords:** Physical activity questionnaire, ActiGraph accelerometers, test-retest reliability, convergent validity, Euclidean Norm Minus 1g (ENMO), young children

## Abstract

**Background:**

The Movement Behavior Questionnaire-Child (MBQ-C) is a caregiver-reported tool designed to assess 24-hour movement behaviors (physical activity [PA], sleep, and screen time) in children under age 5 years who are able to walk. This study evaluated the test-retest reliability and convergent validity of the MBQ-C PA domain among toddlers aged 1–2 years.

**Method:**

This ancillary study was conducted within the Child and Mother Physical Activity Study (CAMPAS), a longitudinal cohort examining early childhood PA development. Toddlers aged 1–2 years wore an ActiGraph wGT3X-BT accelerometer on the hip for 7 consecutive days. Mothers completed the MBQ-C PA domain twice, once before and once after the wear. The domain included four items assessing time spent in active play (categorical responses from 0 minutes to > 4 hours per day) and energetic play (categorical responses from 0 minutes to > 2 hours per day) on a typical weekday and weekend day. Responses were converted to minutes per day using the midpoints of categorical ranges. Test-retest reliability was evaluated using intraclass correlation coefficients (ICCs). Convergent validity was examined using Spearman correlations between MBQ-C-derived and accelerometer-derived PA metrics.

**Results:**

A total of 71 toddler-mother dyads were included in analysis (child age = 24 ± 4 months; range = 16–34 months). The proportions of respondents reporting “0 minutes” on the four MBQ-C PA items ranged from 0–10%. No respondents reported the highest response to any MBQ-C PA items, indicating no to minimal floor and ceiling effects. Accelerometer-derived mean acceleration was 21.0 ± 4.5 m*g*. Accelerometer-derived total PA and moderate- and vigorous-intensity PA were 245 ± 42 and 79 ± 23 minutes/day, respectively. Test-retest reliability was poor for active play (ICC = 0.45; 95% CI = 0.20, 0.64) and moderate for energetic play (ICC = 0.57; 95% CI = 0.38, 0.76), respectively. MBQ-C-derived PA estimates were not correlated with accelerometer-derived PA estimates (ρ=−0.18 to 0.07; all *p* > 0.05).

**Conclusion:**

Among toddlers aged 1–2 years, the MBQ-C PA domain demonstrated poor to moderate test-retest reliability and very low convergent validity relative to accelerometer-derived PA measures. These findings underscore the challenges of accurately capturing toddlers’ PA through caregiver report, given the highly sporadic and unstructured nature of movement at this age.

## Introduction

Regular physical activity (PA) supports healthy development and overall well-being in early childhood.^1–5^ The World Health Organization (WHO) recommends that children aged 1–5 years engage in at least 180 minutes of total PA per day, including moderate- to vigorous-intensity activities.^5^ Although accelerometers are widely recognized as valid tools for assessing PA in children,^6–10^ accelerometerbased assessments are costly and often impractical due to participant burden and logistical constraints in large-scale surveillance or clinical settings.^11,12^ Therefore, there is a growing need for a brief, practical tool to evaluate young children’s PA that can be implemented efficiently in population monitoring and clinical practice.^13–15^

Several caregiver-reported PA questionnaires for young children exist;^12^ however, few have been validated specifically for toddlers aged 1–2 years. For example, the Preschool-age Physical Activity Questionnaire (Pre-PAQ)^16^ was developed and validated for preschool-aged children (3–5 years); however, it was not designed for toddlers. The Patient-Reported Outcome Measurement Information System (PROMIS) Early Childhood PA scale was more recently developed to target children aged 1–5 years;^17^ yet, our prior work found low convergent validity and notable floor effects among toddlers.^18^

The Movement Behavior Questionnaire-Child (MBQ-C)^19^ is a newly developed caregiver-reported tool designed to rapidly assess 24-hour movement behaviors (i.e., PA, sleep, and screen time) among children under 5 years who are able to walk independently. The MBQ-C PA domain consists of only 4 items, offering a brief and practical approach well-suited for large-scale studies or clinical settings. Initial validation of the MBQ-C among Australian children demonstrated good test-retest reliability and acceptable convergent validity for the PA domain.^19^ In a subsequent validation study among Chinese kindergarteners, test-retest reliability was only moderate, and convergent validity was acceptable;^20^ however, findings were largely limited to children aged 4–5 years. Given the substantial developmental differences in movement patterns between toddlers and preschool-aged children, further validation in toddlers is essential before broader application in this age group. To fill this research gap, the present study examined the test-retest reliability and convergent validity of the MBQ-C PA domain among toddlers.

## Methods

### Participants

An ancillary study was conducted within the Child and Mother Physical Activity Study (CAMPAS) cohort. CAMPAS is an ongoing longitudinal study examining the development of PA during early childhood.^21,22^ Eligibility criteria for child participants included being 10–15 months old at baseline, residing in the Chicago metropolitan area, and having no diagnosis of cerebral palsy or other medical conditions that could limit mobility. Mothers were eligible if they were aged 18 years or older, lived with the child at least 50% of the time, and spoke either English or Spanish. Participants were recruited through community flyers and electronic outreach. CAMPAS assessments were conducted in person or remotely at sixmonth intervals. Detailed study procedures have been described previously.^21^

Between May and October 2024, CAMPAS participants were invited to participate in this ancillary MBQ-C validation study. The study protocol was approved by the Institutional Review Boards of Ann & Robert H. Lurie Children’s Hospital of Chicago and Northwestern University. Written informed consent was obtained from all participating parents.

### Measurements

#### Movement Behavior Questionnaire-Child (MBQ-C) Physical Activity Domain.

The MBQ-C PA domain includes 4 items asking caregivers to think about the past week and report the amount of time their child engaged in active play on a typical weekday (Q1A) and a typical weekend day (Q2A), and in energetic play (worded as “vigorous activities”) on a typical weekday (Q1B) and weekend day (Q2B). Vigorous activities were described as running, jumping, dancing, and riding bikes or scooters.

Of the two available MBQ-C versions (open-ended and closed-ended), we used the closed-ended version, because its rapid scoring allows for immediate feedback, making it a practical option for clinical and primary care settings.^19^ Response categories for active play were: 0 minutes, 1–30 minutes, 30–60 minutes, 1–2 hours, 2–3 hours, 3–4 hours, and more than 4 hours per day. Response categories for energetic play included: 0 minutes, 1–15 minutes, 15–30 minutes, 30–60 minutes, 1–1.5 hours, 1.5–2 hours, and more than 2 hours per day.

To assess test-retest reliability, mothers were instructed to complete the MBQ-C PA domain twice: first via an online survey link sent before their child’s accelerometer wear period, and again using a paper version on the final day of wear. Responses were converted to minutes per day using the midpoint of each categorical range (e.g., 0 minutes for “0 minutes”; 15 minutes for “between 1 and 30 minutes”; 45 minutes for “between 30 and 60 minutes”; 90 minutes for “between 1 and 2 hours”).^19^ Daily active play and energetic play minutes were computed as (weekday minutes × 5 + weekend day minutes × 2) ÷ 7.^19^

#### Accelerometer assessment.

To examine convergent validity, we used ActiGraph wGT3X-BT accelerometers (100 Hz; Ametris LLC; Pensacola, Florida, USA). Children wore the device on their hip for 7 consecutive days. Accelerometer data were downloaded and integrated into a 15-second epoch using the ActiLife software (version 6.14). Two data processing approaches were applied. First, the ActiLife software was used to estimate cut-point based PA levels. Data recorded between 6:00 AM and 10:00 PM were extracted.^23–25^ Non-wear time, defined as ≥ 20 consecutive minutes of zero counts,^26–29^ was excluded. A valid day required ≥ 480 minutes of wear time within the 16-hour window.^26,30,31^ Participants with ≥ 4 valid days were included in analysis.^31^ For intensity classification, we applied toddler-specific cut-points^6^ rather than the random forest algorithm^32^ used in the original MBQ-C validation,^19^ because that algorithm has not been validated for toddlers. Light-intensity PA (LPA) was defined as 25–417 vertical counts per 15 seconds, and moderate- and vigorous-intensity PA (MVPA) as > 417 counts per 15 seconds.^6,28^ Daily minutes of MVPA and total PA (LPA + MVPA)^27,28^ were computed for each participant.

Second, data were processed using the GGIR R package^33,34^ to compute Euclidean Norm Minus 1*g* (ENMO).^35,36^ ENMO is a device-independent metric that provides a robust, comparable measure of movement intensity that is independent of device cut-points, allowing for cross-study comparisons. Non-wear periods were identified using GGIR’s default settings, which detects sustained periods of low acceleration variability. Missing values during non-wear periods were imputed using GGIR’s built-in imputation procedure.^33,34^ Mean ENMO values were averaged across valid days to compute mean acceleration (m*g*), representing overall PA volume.^36^

#### Other measurements.

Mothers reported demographic and household information, including the child’s sex, age, race, ethnicity, maternal education, and childcare attendance. Residential addresses were used to assign a Child Opportunity Index (COI) score, a validated indicator of neighborhood resources and conditions relevant to a child’s health and development.^37^ COI scores were categorized into five levels: very low, low, moderate, high, and very high. WHO weight-for-length percentiles were calculated^38^ using anthropometric data obtained from the child’s most recent clinic visit summary.

### Statistical Analysis

All statistical analyses were conducted using SAS 9.4 (SAS Institute; Cary, North Carolina, USA). Descriptive analyses were performed for all study variables. PA variables were summarized and stratified by child age (1 vs. 2 years). Frequency distributions were examined to identify the proportion of participants who selected “0 minutes” (“floor”) and those who selected the maximum response (“more than 4 hours per day” for active play or “more than 2 hours per day” for energetic play; “ceiling”).^17^ Floor and ceiling effects were considered acceptable if ≤ 15% of participants endorsed the lowest or highest response category.^39^

Test-retest reliability for MBQ-C-derived daily active-play and vigorous-activity minutes was evaluated using intraclass correlation coefficients (ICCs) among those who completed the retest within 14 days after the initial test. Convergent validity was examined using Spearman rank correlation coefficients (ρ) between MBQ-C-derived PA estimates at retest and accelerometer-derived PA estimates. Retest data were used to align the MBQ-C’s “past week” recall period with the accelerometer wear period.

A priori power calculations^40^ indicated that a sample size of 36 would provide 80% power to detect ICC ≥ 0.75 (null ICC = 0.50) using F-test at a significance level of 0.05 (two-sided). For convergent validity analysis, a sample size of 51 would provide 80% power to detect a Spearman correlation coefficient ρ ≥ 0.40 between MBQ-C-derived and accelerometer-derived PA estimates under the null hypothesis ρ = 0.00 at a significance level of 0.05 (two-sided).

## Results

A total of 72 mother-child dyads were enrolled in this ancillary study. One dyad was excluded due to insufficient accelerometer data and no retest MBQ-C data, resulting in 71 dyads for analysis. Children had a mean age of 24 (standard deviation [SD] = 4) months (range: 16–34 months), and all were able to walk independently. Mothers had a mean age of 35 (SD = 4) years (range: 23–41 years). The average accelerometer wear time was 14.9 (SD = 1.3) hours/day within the 6 AM-10 PM window. Accelerometer-derived mean acceleration was 21.0 (SD = 4.5) m*g* (Table 1). Means of total PA and MVPA were 245 (SD = 42) and 79 (SD = 23) minutes/day, respectively. As shown in Table 2, accelerometer-derived PA estimates tended to be lower among 1-year-olds than 2-year-olds (*p* = 0.03–0.13). In contrast, MBQ-C PA estimates tended to be higher among 1-year-olds than 2-year-olds (*p* = 0.07–0.19).

### Test-retest reliability.

Test-retest reliability was evaluated among 64 participants, excluding 7 whose test-retest interval exceeded 14 days. Among the 64 included participants, the test-retest interval ranged from 4 to 14 days (median of 7 days; interquartile range of 4–9 days). Among those who completed the retest within 7 days, the ICC was 0.45 (95% confidence interval [CI] = 0.23, 0.67) for active play minutes and 0.58 (95% CI = 0.40, 0.77) for energetic play minutes (Table 3). Among those who completed it within 14 days, the ICC was 0.32 (95% CI = 0.13, 0.52) for active play minutes and 0.58 (95% CI = 0.43, 0.73) for energetic play minutes.

### Validity.

**Figure 1.**
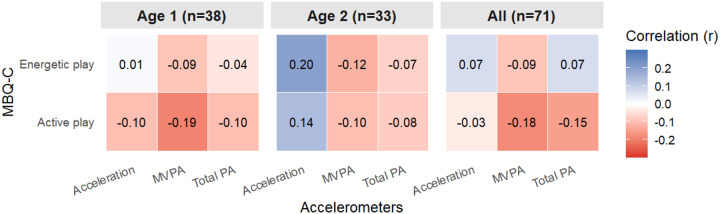
Correlations of MBQ-C and accelerometer measures

The proportions of respondents endorsing “0 minutes” for Q1A, Q1B, Q2A, and Q2B were 1%, 9%, 0%, and 10%, respectively. No respondents selected the highest category (“more than 4 hours” for active play or “more than 2 hours” for energetic play). In the convergent validity analysis, MBQ-C-derived active and energetic play estimates were not significantly correlated with accelerometer-derived mean acceleration, total PA, or MVPA (*p* > 0.05; Fig. 1).

## Discussion

This study examined the test-retest reliability and convergent validity of the MBQ-C PA domain among toddlers aged 1–2 years. Energetic play demonstrated moderate test-retest reliability (ICC = 0.58), whereas active play showed poor test-retest reliability (ICC = 0.45). No to minimal floor and ceiling effects support the measure’s feasibility for use in this age group. However, correlations between MBQC-derived PA estimates and accelerometer-derived PA metrics were negligible, indicating limited convergent validity of the MBQ-C PA domain for toddlers.

### Comparison of PA levels with prior MBQ-C validation studies.

In this toddler sample, MBQ-C-derived active play estimates (107 minutes/day at retest) were lower than those reported in the previous MBQ-C validation studies: 193 minutes/day in an Australian sample (n = 54)^19^ and 128 minutes/day in a Chinese kindergarten sample (n = 211).^20^ Similarly, MBQ-C-derived energetic play estimates (35 minutes/day) were lower than 75 minutes/day^19^ and 69 minutes/day^20^ observed in those respective samples. Accelerometer-derived PA levels in our study (245 minutes/day of total PA; 79 minutes/day of MVPA) were also substantially lower than the Chinese sample (approximately 500 minutes/day of total PA; 250 minutes/day of MVPA), despite our use of lower cut-points applied to hip-worn accelerometer data. Direct comparisons with the Australian sample are limited due to different wear locations (hip vs. wrist). While the CAMPAS cohort is considered relatively active compared with toddler samples in the US^31^ and Australia^23^ and with estimates from meta-analysis,^41^ its PA levels remained far below those observed in prior MBQ-C validation studies of older children, highlighting age-related differences in daily movement patterns between toddlers and preschool-aged children.

### Comparison of test-retest reliability with prior MBQ-C validation studies.

Our test-retest reliability estimates (ICC = 0.45–0.58) were markedly lower than those reported in the Australian sample by Trost et al. (ICC = 0.86 to 0.88)^19^ and somewhat lower than those reported in the Chinese sample by Song et al. (ICC = 0.52 to 0.66).^20^ The lower ICCs in our study could partly be explained by a longer test-retest interval. Trost et al.^19^ administered both the test and retest after accelerometer wear within a short 3-day interval, whereas both our study and Song et al.^20^ conducted the initial test before accelerometer wear and the retest after a 7-day or longer interval. Additionally, the lower ICCs could also be explained by significantly lower PA levels reported at retest as compared to the initial test. Because the retest occurred approximately 7 days after the initial test, it is possible that children were genuinely less active during the retest week. Another potential explanation is that mothers may have reduced over-reporting at retest due to increased awareness that their child’s PA was monitored with an accelerometer during that period. These design differences likely contributed to the lower test-retest reliability observed in our study compared with Trost et al.^19^

### Convergent validity.

This study found that correlations between MBQ-C-derived and accelerometer-derived PA were negligible, in contrast to prior findings from Trost et al. (ρ = 0.25–0.39)^19^ and Song et al. (ρ = 0.35).^20^ The discrepancy may stem from the challenges conceptualizing toddlers’ active play and energetic play and estimating their play durations. At this age, PA often consists of brief, intermittent bursts rather than discrete and sustained activity bouts.^42,43^ This activity pattern makes it challenging to estimate intensity and duration of PA.^42,43^ Consistent with developmental expectations, accelerometer-derived PA levels were higher among 2-year-olds than 1-year-olds; however, MBQ-C-derived PA estimates showed the opposite pattern, resulting in small, but nonsignificant, negative correlations (e.g., ρ = −0.18 between MBQ-C-derived active play and accelerometer-derived MVPA). This mismatch illustrates inherent difficulties caregivers face in estimating intensity and duration of toddler movement.

### Challenges in caregiver-reported PA assessment for toddlers.

In response to the growing need for brief, practical PA assessment tools in young children, new caregiver-reported instruments have been recently developed, including PROMIS Early Childhood PA scale.^17^ The PROMIS PA scale is distinctive in that it captures children’s physiological responses to PA and quantifies the number of active days per week (e.g., “In the past 7 days, how many days did your child so physically active that he/she sweated?”; “How many days did your child play so hard that he/she felt tired?”^17^) rather than the amount of daily PA. Our validation of the PROMIS Early Childhood PA scale among toddlers found moderate test-retest reliability and poor convergent validity with accelerometer measures.^18^ Unlike PROMIS EC, the MBQ-C quantifies daily duration of active and energetic play, aligning more directly with WHO 24-hour movement guidelines^5^ and the emerging 24-hour movement behavior paradigm,^44^ thereby offering conceptual advantages for surveillance. Nonetheless, the present findings indicate that among 1- to 2-year-olds, the MBQ-C PA domain demonstrates only modest reliability and poor correspondence with accelerometer-derived measures. Subjective recall of intensity and duration is prone to estimation bias without clear benchmarks for what constitutes “active” and “vigorous” play among toddlers.^42^ Rapid PA assessment among toddlers may need to incorporate alternative proxy indicators, such as PA environments and parenting practices.^45^

### Limitations.

This ancillary study leveraged the existing CAMPAS protocol, in which the initial MBQ-C administration occurred before accelerometer deployment. In addition, the test-retest interval was longer than optimal for reliability evaluation. These study design aspects may have contributed to the lower ICCs. Second, we evaluated only the PA domain of the MBQ-C; because 24-hour time-use diary data were unavailable we could not evaluate the validity of the screen time or sleep domains. Finally, the study relied exclusively on maternal reports and fathers were not included, which may limit the generalizability of findings to broader caregiver contexts.

## Conclusions

Among children aged 1–2 years, the MBQ-C PA domain demonstrated poor-to-moderate test-retest reliability and very low convergent validity relative to accelerometer-derived PA metrics. These findings underscore the inherent challenges of caregiver-reported PA assessment in toddlers whose movement patterns are highly sporadic and unstructured. Rapid PA assessment among toddlers may need to incorporate alternative proxy indicators, such as PA environments and parenting practices. While the MBQ-C PA domain has shown acceptable performance in preschool-aged children, its use for toddlers needs further validation in population-based samples across diverse counties and cultural contexts. Adaptation or modification of the MBQ-C PA domain may be necessary before it can be applied for PA assessment among children aged 1–2 years.

## Figures and Tables

**Table 1:** Participant characteristics

Variable	n (%)
Total	71 (100%)
Sex	
Female	39 (45)
Male	32 (55)
Age	
1 year	38 (54)
2 years	33 (46)
Maternal education	
< 4-year college degree	8 (11)
≥ 4-year college degree	63 (89)
Child Opportunity Index	
Very low or low	18 (25)
Moderate	16 (23)
High or very high	37 (52)
Race and ethnicity	
Hispanic	13 (18)
Non-Hispanic Black	6 (8)
Non-Hispanic White	41 (58)
Non-Hispanic multi-race	5 (7)
Others	6 (8)
Childcare attendance	
0 days	32 (45)
1–3 days/week	8 (11)
4–5 days/week	31 (44)
Accelerometer-derived variables	mean ± SD
Mean acceleration, m*g*	21.0 ± 4.5
Total PA, minutes/day	245 ± 42
MVPA, minutes/day	79 ± 23

MVPA, moderate- and vigorous-intensity physical activity; PA, physical activity; M ± SD, mean ± standard deviation.

**Table 2:** Accelerometer-derived and MBQ-C-derived physical activity variables by age

	1-year-olds (n = 38)	2-year-olds (n = 33)	Comparison by age
	M ± SD	M ± SD	T-test p-value
Mean acceleration, m*g*	20.2 ± 4.3	21.9 ± 4.7	0.13
Total PA, minutes/day	239 ± 41	258 ± 46	0.06
MVPA, minutes/day	73 ± 18	85 ± 27	0.03
MBQ-C active play, minutes/day	39 ± 30	30 ± 27	0.19
MBQ-C energetic play, minutes/day	119 ± 59	92 ± 63	0.07

M ± SD, mean ± standard deviation; MBQ-C, Movement Behavior Questionnaire-Child; MVPA, moderate- and vigorous-intensity physical activity; PA, physical activity.

**Table 3:** Test-retest reliability for the MBQ-C physical activity domain

	Test (n = 64)		Retest (n = 64)		Retest within 7 days (n = 41)	Retest within 14 days (n = 64)
	M ± SD	Median (IQR)	M ± SD	Median (IQR)	ICC (95% CI)	ICC (95% CI)
Active play, minutes/day	138 ± 54	150 (90, 210)	107 ± 62	90 (58, 150)	0.45 (0.23, 0.67)	0.32 (0.13, 0.52)
Energetic play, minutes/day	41 ± 29	29 (23, 29)	35 ± 29	23 (12, 45)	0.58 (0.40, 0.77)	0.58 (0.43, 0.73)

CI, confidence interval; ICC, intraclass correlation coefficient; IQR, interquartile range; M ± SD, mean ± standard deviation; MBQ-C, Movement Behavior Questionnaire-Child

MBQ-C, Movement Behavior Questionnaire-Child; MVPA, moderate- and vigorous-intensity physical activity; PA, physical activity

## Data Availability

Data may be available upon request.

## Abbreviations

CI: confidence interval
ICC: intraclass correlation coefficient
LPA: light-intensity physical activity
MBQ-C: Movement Behavior Questionnaire-Child
MVPA: moderate- and vigorous-intensity physical activity
PA: physical activity
PROMIS: Patient-Reported Outcome Measurement Information System
SD: standard deviation
